# An Overview on Wood Waste Valorization as Biopolymers and Biocomposites: Definition, Classification, Production, Properties and Applications

**DOI:** 10.3390/polym14245519

**Published:** 2022-12-16

**Authors:** Francesca Ferrari, Raffaella Striani, Daniela Fico, Mohammad Mahbubul Alam, Antonio Greco, Carola Esposito Corcione

**Affiliations:** Department of Engineering for Innovation, University of Salento, Via Arnesano, 73100 Lecce, Italy

**Keywords:** biopolymers, biocomposites, renewable sources, wood waste, waste valorization

## Abstract

Bio-based polymers, obtained from natural biomass, are nowadays considered good candidates for the replacement of traditional fossil-derived plastics. The need for substituting traditional synthetic plastics is mainly driven by many concerns about their detrimental effects on the environment and human health. The most innovative way to produce bioplastics involves the use of raw materials derived from wastes. Raw materials are of vital importance for human and animal health and due to their economic and environmental benefits. Among these, wood waste is gaining popularity as an innovative raw material for biopolymer manufacturing. On the other hand, the use of wastes as a source to produce biopolymers and biocomposites is still under development and the processing methods are currently being studied in order to reach a high reproducibility and thus increase the yield of production. This study therefore aimed to cover the current developments in the classification, manufacturing, performances and fields of application of bio-based polymers, especially focusing on wood waste sources. The work was carried out using both a descriptive and an analytical methodology: first, a description of the state of art as it exists at present was reported, then the available information was analyzed to make a critical evaluation of the results. A second way to employ wood scraps involves their use as bio-reinforcements for composites; therefore, the increase in the mechanical response obtained by the addition of wood waste in different bio-based matrices was explored in this work. Results showed an increase in Young’s modulus up to 9 GPa for wood-reinforced PLA and up to 6 GPa for wood-reinforced PHA.

## 1. Introduction

Nowadays, the manufacturing of bio-based polymers is characterized by a strong development. Because of the present rapid expansion in the manufacture of these polymers, the use of plastic items created from them is also expanding, according to the European Environment Agency (EEA) [1]. However, they even correspond to a very modest portion of the market, since they are around one percent of the more than 368 million tons of plastic produced yearly. The total amount of biopolymers is going to increase up to 2.11 million tons [2]. The main challenge in the next few years will be to significantly reduce production costs. Economies of scale are vital for competitive pricing. However, because bio-based supply chains are often extensive, scaling them up is difficult, especially since many of the essential technologies have not been validated [3,4].

Today, growing attention is paid to the manufacturing of bio-based materials, starting from scraps. The circular economy has, in fact, grown in importance in academic study over the previous decade. A key development goal of the circular economy is the reuse of different kinds of wastes, particularly wastes from industrial operations [5]. The increase in population and the usage of polymers for disposable items and packaging generate uncontrolled waste, posing severe management and disposal issues. The unrestricted waste stream from many sources poses a major challenge to waste management [6]. Several researchers have been studying the potential and valorization of the organic component in a circular economy scenario. The valorization technique has numerous benefits over conventional organic fraction collecting and treatment technologies. These treatments use the organic portion as an energy source by burning or composting it [7,8].

The organic fraction of municipal scraps includes a huge quantity of carbohydrate and wood derivatives [9]. In today’s world, the increase in population and the continuous usage of throwaway materials for lots of applications causes significant problems in waste management [2].

Among the different kinds of organic wastes, agro-industrial and forestry wastes present an unsustainable environmental and economic scenario [10]. Therefore, the valorization of this class of wastes represents one of the main issues in terms of disposal management. Many studies are currently being carried out to develop new procedures to produce biopolymers and biocomposites, starting with agro-industrial wastes. Due to the innovation of the topic, a summary of the last developments in the production of biopolymers and biocomposites can be useful to help carry out future research.

This work reports the current developments in the classification, production, properties and application of biopolymers, particularly those obtained from the valorization of wood wastes. Among the different application fields, the possibility to use wood scraps as a reinforcement for the production of biocomposites is also reported and the improvements in the performances obtained by the use of wood waste fillers are analyzed.

## 2. General Definition of Biopolymers and Biocomposites

### 2.1. Biopolymers: Difference between Biodegradable and Bio-Based

A polymer can be defined as a bioplastic when the material exhibits biodegradability, or it comes from bio-based or raw materials, or both [11,12]. Other researchers define a bioplastic as a material that can be decomposed into CO_2_, H_2_O and non-organic particles or biomass, generally thanks to the enzymatic decomposition carried out by microorganisms. Nevertheless, a bio-based polymer could be not biodegradable and vice versa. A bio-based polymer is derived from natural sources obtained from biomass, which can be partially or totally renewable. There are three basic methods for creating bio-based polymers. To achieve the performance criteria, one option is to partially change polymers derived from green sources, for example, cellulose, and lipids, using extraction, separation and filtration [13].

Depending on the type of synthesis and source, biopolymers can be classified into three groups (Table 1) [14]. Natural sources, such as carbohydrates and proteins, or monomers, such as lactic acid, can be the raw materials for biopolymers. Furthermore, other biopolymers such as polyhydroxyalkanoate (PHA) can be obtained from microorganisms [15]. Commercially accessible biopolymers are divided into the categories which follow: polyhydroxyalkanoates (PHAs/PHBs), polylactic acid (PLA) polyamides, polyols, bio-PET, butyl rubber and cellulose acetate; these are some of the materials used [16]. For practical usage in plastics or as water-soluble polymers, polysaccharides are mostly confined to starch and cellulose derivatives. Both of these compounds are made up of D-glycopyranoside-repeating units, which result in molecular weights in the thousands [14].

#### 2.1.1. Biodegradable Polymers

Biodegradability is an important property of biopolymers, which does not refer to the raw materials used for the production. A biodegradable polymer could, in fact, be derived from fossil sources. Biodegradation is a biological process that occurs during composting, which involves the release of carbon dioxide, water, non-organic particles and biomass. In order to be defined as biodegradable, the biopolymer should degrade with a similar rate of certified compostable materials, without leaving hazardous residue [25]. If the plastic is able to decompose, but it doesn’t follow the established standards, even if it is biodegradable, it must be classified as non-compostable. Finally, degradable materials should also not be derived from natural sources; for example, oxo-biodegradation is a phenomenon that occurs with some polyolefins through an oxidative process, which implies the breakage of the plastics into small pieces making them easier to biodegrade. Nevertheless, oxo-degradation is not currently classified as biodegradable or compostable, as its decomposition does not occur following established standards [26].

Table 2 shows the biodegradability of the most-used bioplastics in different fields. Biopolymer degradation depends on the physical and chemical structure of the biomaterial [27]. Furthermore, pH, temperature, moisture and oxygen must be considered.

#### 2.1.2. Bio-Based Polymers

The greatest part of bioplastics currently available on the market are obtained from biomasses of the first generation, for example, corn, potatoes, sugar cane, palm oil and straw. All these sources have a high amount of carbohydrates and can be eaten by people and animals. Feedstocks of the first generation have a high efficiency for the production of biopolymers, since they need less land to grow and have a high yield of production compared to the other feedstocks. The technical maturity of these feedstocks is then very high [25], although the subtraction of sources to the food chain poses important issues.

The second generation of feedstocks is related to those raw materials which cannot be eaten by animals, such as non-edible harvests (e.g., cellulose) or derivatives of raw materials of the first generation, for example, sugarcane bagasse. Although second-generation feedstocks are commercially available, the use is not so widespread due to a relatively high cost.

Finally, the third-generation feedstock, obtained from food scraps, algae biomass and industrial or municipal waste, is the most innovative and can solve the problems related to the consumption of sources from the food chain. Several studies are underway in order to develop new biopolymers from food wastes [31].

**Figure 1:**
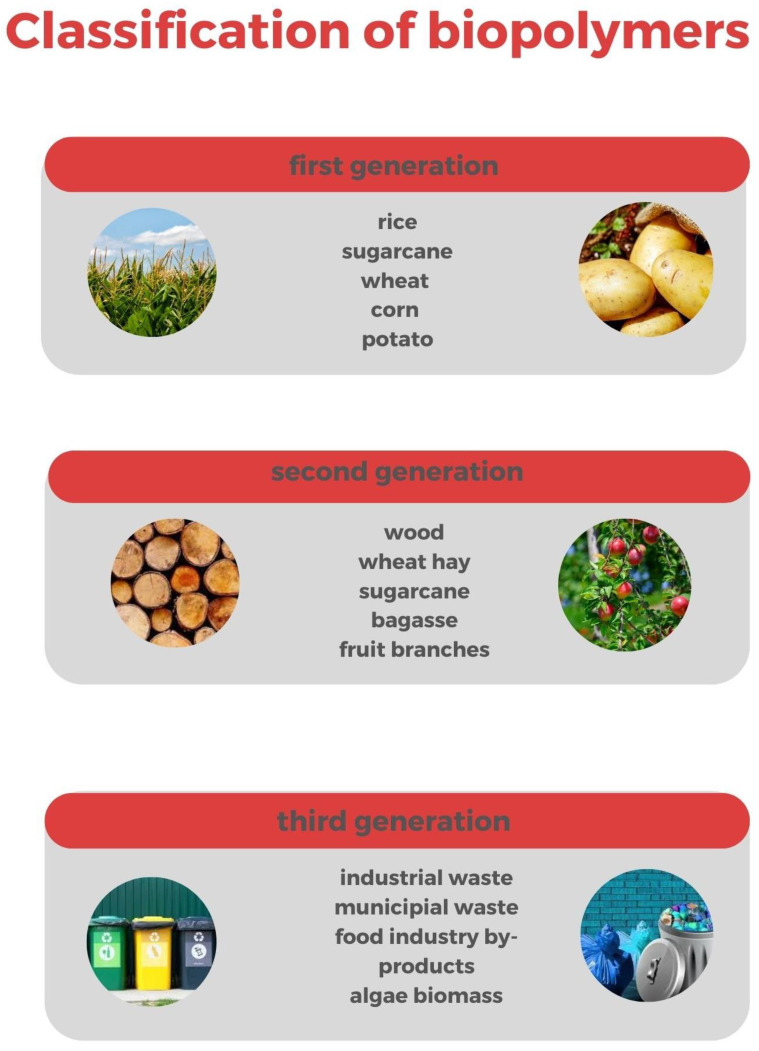
An overview of the classification of biopolymers.

Figure 1 shows a summary of the three generations of feedstocks.

This review is focused on a special class of the third-generation biopolymers, i.e., those obtained from the reuse and valorization of wood-based waste. The following paragraphs, hence, refer to the description of the main production methods, properties and applications of biopolymers from wood sources, with a special focus on wood scraps, such as agricultural waste.

### 2.2. Biocomposites

The tendency to substitute the petroleum-based polymers with biopolymers has also made its way into the field of composite materials. In the last decade, it has become a possibility to combine biopolymers with natural fillers in developing new materials, i.e., biocomposites. They have attracted the interest of the research community all over the world, as well as the industrial sector, for a wild spectrum of applications that these new materials can offer, such as aerospace parts, automotive components, consumer goods, sporting goods and their use in the marine and oil industries [32]. Biocomposites are defined as composite materials composed of biodegradable natural fibers used as reinforcement and biodegradable (or non-biodegradable) polymers in a matrix. Starch, cellulose, soya, polylactic acid and polyhydroxyalkanoates are the most commonly available biopolymers [33]. Natural fibers are largely divided into two categories: plant-based and animal-based. In general, plant-based fibers are lignocellulosic in nature, and they are composed of cellulose, hemicellulose and lignin; animal-based fibers consist of proteins, for example, silk and wool [34]. In fact, the great advantage offered by biocomposites with respect to traditional composites is related to low energy and low CO_2_ emission during their processing, biodegradability, renewability, low specific weight, higher specific strength, and stiffness, high electrical resistance, low cost and good thermal and acoustic insulating properties [35]. Despite some drawbacks that affect biocomposites, mostly due to their high sensitivity to moisture, low durability and low adhesion between matrix and fiber [36], natural fillers play an important role in order to develop fully biodegradable green composites as a possible solution for contemporary environmental difficulties.

## 3. Production of the Main Biopolymers from Bio-Based Sources

### 3.1. Cellulose Traditional Sources

Cellulose is constituted of anhydroglucose units linked by a β-(1,4) glycosidic bond. The repeating unit of cellulose is the glucose dimer known as cellobiose. In the condensation process, glycosidic oxygen bridges the sugar rings which are formed and the cellulose chains reach a degree of polymerization (DP) around 15,000 in native cellulose cotton and 10,000 glucose units in wood cellulose. Each monomer has three hydroxyl groups that allow the creation of hydrogen bonds by influencing the crystalline packing and, consequentially, the cellulosic physical characteristics. The van der Waals and intermolecular hydrogen interactions allow numerous cellulose chains to stack in parallel and self-assemble into microfibrils, constituted of crystalline sections in which the cellulose chains are organized in a highly ordered form, and amorphous regions, which are less ordered than the former [37,38]. For such a reason, cellulose is a semi-crystalline substance whose crystallinity is determined by its source, extraction technique and treatments. The degree of crystallinity of wood-based and plant-based cellulose is typically 40–60% [39].

Cellulose is a nearly limitless polymeric raw material since it is the most abundant component in most plants. The availability, renewability and biodegradability of this material, as well as its low cost, are the major benefits, but it has two major drawbacks when compared to synthetic polymers: high hygroscopicity, owing to hydroxyl groups, and limited processing due to rapid disintegration [40]. Cellulose can be extracted from several natural sources, such as wood (lignocellulosic biomass), agricultural scraps, cotton, flax, hemp, sisal and especially vegetable byproducts [40]. Wood pulp is the most widespread raw material for cellulose processing, especially for paper and cardboard manufacture [31,41,42,43].

It is well known that cellulose, which is extracted from wood such as spruce, pine and many other trees, is being used to produce regenerated fibers such as viscose, lyocell, modal, cellulose acetate and cellulose triacetate. Cellulose esters and ethers are the principal industrially used cellulose derivatives, with the former being used in molding, extrusion and films, and the latter in a varied range of application fields (building materials, food, personal care products, paints and pharmaceuticals). However, until solvent methods for dissolving cellulose became available, the processability and application of this type of cellulose in biodegradable plastic films were limited. In order to modify the mechanical and chemical properties of cellulose, plasticization and blending with other polymers are used [44]. There have been a number of recent developments in the field of polymeric thermoplastic film and functional polymeric materials such as composite and composite films [45]. Cellulose-based biocomposite systems use cellulose as a reinforcement and/or matrix (host material) [45]. Cellulose fibers and derivatives are currently being used to make biopolymeric materials such as fillers and polymer matrices in biopolymer composites. Recent biocomposite research has enabled the replacement of petroleum-based polymers (PE and PP) with naturally generated biopolymers, such as cellulose and starch, and glass fibers with cellulose fibers.

Cellulose could also be used in wastewater treatment because it is naturally hydrophilic, it has been employed as an antifouling hydrophilic coating on membranes in order to increase the flow of the membranes and also the adsorption capability of cellulose-based functional materials is excellent for water treatment applications. It has been found that cellulose has a high adsorption capacity for pollutants after being subjected to appropriate chemical alteration on its surface, with the goal being the absorption of molecules with basic groups, particularly those containing significant concentrations of nitrogen, sulfur and oxygen [45]. In fact, as reported by Li et al. [46], cellulose, as well as other biopolymers such as lignocellulose, chitosan, chitin and lignin, shows a good absorption capability of heavy metal ions from aqueous solutions. Li et al. [46] collected the main studies related to cellulose properties, in particular, the capability of a cellulose-based copolymer to adsorb chromium (VI) and convert it in Cr (III) by means of the ultrasonication method [47]; the capability of cellulose aerogels to encapsulate iron oxides [48] and the employment of carboxylated cellulose derivatives for absorbing Co^2+^, Cu^2+^ and Ni^2+^ in aqueous solutions [49]. Dassanayake et al. [50] reported the use of cellulose-based materials and derivative (chitin and chitosan)-based materials in specific applications in which their absorption properties are employed, such as the removal of organic dyes and heavy metals, oil and solvent spillage cleanup and CO_2_ adsorption. Furthermore, cellulose-based functional materials are widely employed in biomedical fields such as drug delivery systems [51,52,53]; in cancer therapy [54]; in bone regeneration [55,56] and in tissue engineering [57]. Demitri et al. [58], in fact, developed an innovative method for producing cellulose-based (CMCNa) foams, demonstrating an excellent biocompatibility profile with a good cell proliferation rate. When cellulose is linked with conductive polymers, it can form nanocomposites with high conductivity [59,60]. Shahbazi et al. [61] demonstrated that CMC modified by both photo and chemical cross-linking can improve surface hydrophobicity, the water barrier and mechanical properties of food packaging materials.

The applications of cellulose in the field of sensing material was also studied, including employing cellulose as a membrane for inkjet printing [62]; as CNT–cellulose composites on ammonia sensors [63]; as hybrid cellulose hydrogel used in release systems [64]; and as active mesoporous cellulosic materials with potential applications in optics, tissue engineering, chiral separation, functional membranes and biosensing [65]. Li et al. [46] also reported the employment of cellulose nanocrystal (CNC)–polymer nanocomposites as reinforcing material [66] or RGO–cellulose composites employed in storage energy filed as supercapacitors [67]. Furthermore, in the field of solar cells, cellulose-based composites were found to have a collocation, as studied by Bisconti et al. [68], that realized semi-transparent perovskite–polymer composites by employing hydroxyethyl cellulose and obtaining advantages in terms of ease of processing; improvement of visible transmittance; and enhancement of thermal stability, by preserving the photovoltaic performances of semi-transparent perovskite solar cells.

### 3.2. Lignin Traditional Sources

After cellulose, lignin is the second most predominant sustainable bioresource and it is found in abundance in wood, which is the world’s primary supply. It is considered as a waste product in a number of industrial processes [69,70,71]. Because lignin is found in biomass combined with cellulose and hemicellulose, it serves as a restrictive issue in the bioconversion of wood, which is now under investigation [72,73]. Lignin is a naturally occurring component of wood and plant cell walls. Its polyphenolic chemical structure has been studied for industrial applications. Various delignification chemical procedures can extract lignin from wood, which has a structure and qualities unique to each plant species. In recent years, however, the chemical industry has concentrated on using lignin as a feasible renewable source for the production of innovative and ecological biomaterials. Its organization is complicated and hard to describe, making it difficult to blend into polymers, fibers and other materials [74]. Its hydrophilicity, polyanionic structure and nontoxicity make it an ideal choice for modifying membrane bulk and surface properties. Various lignin derivatives have been studied extensively for bulk modification of polymeric membranes. A preliminary material, such as the wood from which the pulp is made, is normally required for the extraction of lignin from several types of biomass. Lignin yield is influenced by numerous factors, including extraction process, reaction time, medium and temperature. The extraction of lignin from cellulosic pulp can be conducted via enzymatic, chemical and physical techniques. Acid hydrolysis, the kraft process, the lignosulphonate process and organo solvolysis are just a few of the chemical processes used to extract lignin. Acid hydrolysis is a process in which the lignin in wood pulp is dissolved using a mixture of argon and concentrated hydrochloric acid (HCl) [75]. The lignosulphonate procedure includes heating wood with sodium sulfite (aqueous) in acidic conditions to extract lignin. Functions include surfactants, additives, dispersants and flocculants [76].

Lignin is another cheap, renewable, biodegradable plastic preference, which improves matrix polymer compatibility and UV stability. Functional groups in matrix and lignin interact to create positive compatibility with both natural and manufactured polymers. The reinforcing activity and thermal stability of lignin have resulted in a high modulus value. Additionally, thermal insulation ratings are a bit higher than those of other materials [77].

Lignin has been extensively explored for its possible use as a sustainable alternative to petroleum derivatives and chemicals. Its polyphenolic structure makes it suitable for usage in phenolic monomers, polyurethane foams, polyolefins, adhesive resins, packaging materials, unsaturated polyester, epoxy resins and material filler. Lignin can be chemically modified or incorporated into a matrix to provide it with new qualities. Banu et al. [78] selected several pretreatment methods (mechanical, chemical, biological, physical and physiochemical) able to extract lignin from diverse kinds of lignocellulosic biomass and reported the influence on medium and short chains of PHA yield for producing packaging materials, such as films, coatings, bags and bottles [78]. They also reported case studies concerning an increase in the production of PHA using genetically modified and engineered bacteria grown in lignin substrate. Furthermore, Banu et al. reported several synthesis processes aimed at producing lignin nanoparticles and their application in biocide systems [79], drug storage and delivery [80] and coatings [81].

Lignin conversion to high quality products is critical to a biorefinery’s economic success. These studies have established catalytic pathways for the production of aromatic chemical reagents and bio-based compounds, epoxy resins, carbon fibers, phenolic adhesive resins, hydrogels, 3D-printed biocomposite and polyurethane foams [74]. To make polymer matrix composites, the amorphous polyphenolic macromolecule Lignin is utilized as a filler. The composite’s characteristics are enhanced by the inclusion of lignin. Antioxidant properties of lignin make it a good stabilizer for polymers. Because char inhibits the combustion and the heat release rate of polymeric materials, lignin can produce a significant quantity of carbonize residue when heated at a high temperature in an inert atmosphere. This property is fundamental to flame-retardant additives. Lignin can also influence the structure of thermoplastic polymers by acting as a nucleating agent during the crystallization process [82]. A potential alternative to inorganic fillers is represented by lignin-based nanoparticles due to phenolic groups and their UV resistance and antioxidant properties. Furthermore, it was demonstrated that lignin-based nanoparticles are able to improve the mechanical and physical properties of the final nanocomposite. Banu et al. [78] reported the main lignin nanoparticle synthesis processes. Thanks to the several reaction sites of lignin (hydroxyl, carboxylic acid, phenolic clusters) it is possible to cross-link it with the polymeric monomers (polyesters and polyurethanes; phenol–formaldehyde resins). For such a reason, the development of different lignin-based biopolymers is possible. What has been studied, in fact, is how a lignin presence minimizes the biodegradation of polyhydroxyalkanoates (PHA), increasing the resistance towards the microbial activity [83]; how lignin enhances the biodegradability of polyester, increasing the photoreactivity and the glass transition temperature [84]; how lignin is blended to polylactic acid (PLA) in order to obtain higher flame resistance [85]; and how lignin can substitute 30–50% of petroleum-derived polyols for the synthesis process of polyurethane, etc. [86,87].

**Figure 2:**
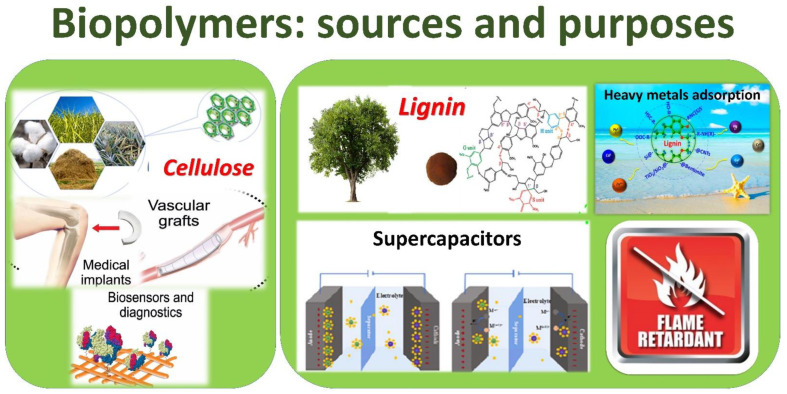
Sources and purposes of biopolymers from traditional wood sources.

Figure 2 reports a schematic overview of the main bio-based polymers from traditional wood sources.

### 3.3. Cellulose and Lignin Biopolymers from Wood Waste

One of the most promising challenges is the possibility of exploiting biomasses from wood as biosources of hemicellulose, cellulose and lignin. Lignocellulosic biomass represents, in fact, the highest amount of unused global biomass [88] and it is mostly composed of dry matter with the addition of oils, minerals and other components, which account for less than 10% [89]. Biomasses from wood wastes can be obtained by using different raw materials, such as forest and crop scraps, municipal solid waste, wood and paper wastes [89,90], which influence the quantity of each constituent of the biomass. Lignocellulosic material shows different amounts of each component, in terms of chemical composition, since they are usually altered by the environment [91]. In particular, based on the amount of biomass, woods can be classified into hardwoods and softwoods which contain, respectively, higher (78.8%) and lower (70.3%) amounts of cellulose and emicellulose and, reversely, there is lower lignin content in hardwoods (21.7%) than in softwoods (29.2%) [89]. Since the cellulose, lignin and hemicellulose amount depends on the kind of wood biomass, a suitable material should be chosen for the fermentation.

Plant biomass can, therefore, be used to produce high-performance functionalized polymers. Large-scale lignocellulosic biomass production will provide plentiful renewable feedstock for biomaterials with physical and chemical performances that are equal to or higher than those of petroleum-based mixtures, including lignin, cellulose and hemicellulosic polysaccharides [92].

Agro-waste recycling by composting and fertilizer manufacturing boosts global carbon emissions, according to data collected in Italy. Wastes from olives (OWC) and anaerobic digester-based compost (ADC), respectively, yielded 64 and 67 kg of CO_2_ equivalent per milligram. Furthermore, each milligram of compost produced by re-composting and co-composting, ranging from 8 to 31 kg of CO_2_, was released [93].

Particular attention was given to agro-waste cellulose for producing biopolymers in a critical review by Motaung et al. [94]. As discussed by the authors, even if several studies on the chemical modification of cellulose fibers were well known, very few discuss agricultural cellulose waste fibers. Sundarraj et al. [95] focused their study on the cellulose derived from agro-industrial residues as effective reinforcement for the building construction material industry. Lately, Urbina et al. [96] collected in their review the main case studies about the production of bacteria cellulose by employing agro-wastes (residues of agricultural products), focusing on the applications of this kind of biopolymer for environmental applications, optoelectronic and conductive devices, food ingredients and packaging, biomedicine and 3D-printing technology. El Achaby et al. [97] studied the employment of red algae waste as a natural resource for producing superior cellulose nanocrystals and their capability to act as strengthening filler.

The extraction of lignocellulose from waste materials represents a great environmental advantage since it avoids the problem that agro-waste can become a source of contamination.

Lignin can also be derived from wood waste. In fact, as demonstrated by Zikeli et al. [98], a lignin fraction was isolated from the wood wastes of a wood house producer for the production of lignin nanoparticles and then used for wood surface treatment. The developed coatings showed significant results after an artificial weathering test. Nevertheless, the extraction of lignin from wood waste is an open research question due to the difficulty of processing and the consideration of lignin as a waste material, i.e., an undesirable component in the manufacture of ethanol and paper, as reported by Parvathy et al. [99]. Thanks to its high thermal stability, biodegradability, antioxidant property, cross-linked structure and UV absorption characteristics, lignin could be effectively employed in several applications to produce valuable materials.

**Figure 3:**
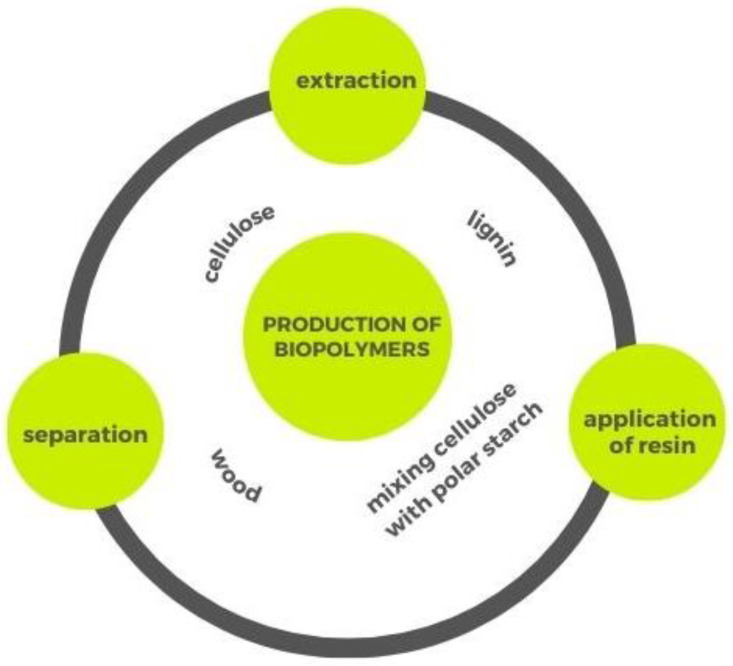
Flow production of bio-based polymers from wood waste.

Figure 3 reports a flow production of bio-based polymers from wood waste.

### 3.4. PHAs’ Traditional Sources

Microbial manufacturing techniques are used to produce polyhydroxyalkanoates (PHAs). PHAs are a class of aliphatic polyester which are naturally obtained in a sugar-based media by bacteria and operate as carbon and energy storage materials. They were the first biodegradable polyesters used in the plastics industry. Aliphatic polyesters are the easiest synthetic polymers to biodegrade [14].

Synthesis of PHAs requires the use of different organisms, especially plants and bacteria, often employed for a large-scale production [100]. On the other hand, plants allow the producing of only small amounts (<10% (*w*/*w*) of dry weight) of PHA, since higher quantities of storage PHAs inside the plants lead to negative effects on the plants’ growth [101]. Therefore, synthesis of PHA is actually carried out by bacteria, which naturally accumulate more than 90% *w*/*w* of PHAs in order to store carbon and energy during the metabolism of nutrients [102]. In particular, the accumulation of PHAs only occurs if bacteria grow with a reduction in oxygen, nitrogen and phosphorous, and an increase in carbon sources [103].

Once the soluble nutrients and intermediates are converted into insoluble PHA polymers, PHAs are stored in intracellular granules inside the cell. In this way, the osmotic state of the cell is preserved, which in turn means a secure storage of the nutrients without any losses through the cell membrane [104].

After the production, the PHA pellets are coated with a layer of proteins and phospholipids. This layer, mainly composed of a particular class of proteins, the phasins, changes both the size and the amount of PHA pellets [105].

**Figure 4:**
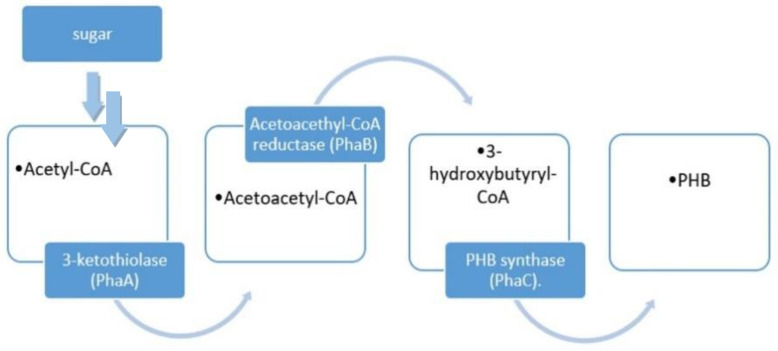
Synthesis pathway of PHB.

Among PHAs, PHB is the first and commonly used, obtained by Alcaligens Eutrophorus bacteria through the conversion of acetyl-CoA in the following three steps [106], reported in Figure 4:

1. First, starting with sugar, two molecules of acetyl-CoA are combined with 3-ketothiolase (PhaA) and acetoacetyl-CoA is obtained.
2. In the second step, acetoacetyl-CoA is reduced using Acetoacethyl-CoA reductase (PhaB) to obtain 3-hydroxybutyryl-CoA.
3. In the third step, PHB is obtained after the conversion of 3-hydroxybutyryl-CoA using PHB synthase (PhaC).

PHB is characterized by high hydrophobicity and can be produced at low temperatures. However, PHB is thermally instable when heated close to the melting of the material. The polymer exhibits essential thermoplasticity and degradability qualities in decomposition and other settings including sea water and, as a result, it has gained a lot of industrial attention [107,108,109]. PHB, a commercially accessible biopolymer, is one of the most attractive members of the polyhydroxyalkanoates family for the packaging of food. PHB is a polymer which should be modified using standard industrial polymer processing facilities. It also has strong mechanical qualities, such as strength and stiffness, that are equivalent to or better than some of the products (such as PP), as well as good barrier properties (similar to PET). PHB degrades in decomposition situations and in other conditions, such as in seawater [109]. Even though PHB is a good choice for green applications such as packaging, it has significant flaws that prevent it from being widely used in the packaging industry. Owing to room temperature crystallization and physiological ageing phenomena, PHB has a relatively high instability which increases over time. PHB also has a small processing range which makes it difficult to treat in some typical packaging applications, such as heat treating [110]. Another major impediment to its application in the packaging industry is its expensive cost, which continues to surpass EUR 5/kg. In this regard, the inclusion of a long-lasting, low-cost or hard filler might help mitigate the raw price rise by (a) minimizing the overall packaging cost and/or (b) decreasing the thicknesses required in standard packaging [110].

### 3.5. PHA from Wood Wastes

A particular problem that limits the use of PHAs is related to its cost of production, starting from the raw materials to the recovery process [111]. In particular, the price of PHA raw materials is strongly attributable to the carbon contribution; therefore, many efforts are currently made in order to replace traditional sources with derivatives of wood scraps [112]. A potential way to replace traditional sources was studied by Kumar [113], who used wood hydrolysates obtained using enzymatic hydrolysis of hemicellulose and cellulose fermentable sugars. To use substrates of wood hydrolysates, the appropriate bacteria must be selected. In his study, Kumar [113] tested various colonies of different morphologies of bacteria for the production of PHA, using biomass substrates obtained from wood wastes. Results obtained allowed the production of PHA from paper paste and tannery effluent water samples with both using gram-positive and gram-negative bacteria, even if gram-negative bacteria were predominant.

PHA properties are mostly related to the length of the polymer chain. Long-chain (PHAs with C ≥ 15), medium-chain (PHAs with 6 ≤ C ≤ 14) and short-chain (PHAs with C ≤ 5) polymers are the three types of PHAs, considering the amount of carbons in the monomer units [114]. Short-chain polymers cannot be used if a high strength is required, because they are too brittle, high-crystalline and stiff. Medium chains have higher elastic modulus and, therefore, show lower brittleness, higher elongation at break and low-crystalline zones. On the other hand, these PHAs are less suited to high-temperature applications [115,116]. Films, fibers [117,118], foams, food additives, medical implants [119], medication delivery carriers, control release material, medical scaffolds for tissue regeneration [120], biofuels [121] and animal feeds all include PHA. PHA can be converted into chiral hydroxyalkanoic acids (HAs). PHA is a renewable substance that should be approved by the market [122]. The high manufacturing costs of PHA, which are at least three times higher than those of traditional materials, e.g., polypropylene (PP), polyethylene (PE) and related biopolymer polylactic acid, have contributed to their limited success [123]; therefore, as explained in previous paragraphs, alternative sources derived from wastes are currently under investigation, which allow for a strong decrease in the production costs.

PHAs showed a higher barrier and mechanical properties than PLA. Nevertheless, they only account for 1.4 percent of the biopolymer industry, even if their manufacturing is expected to double by 2023. PHA has comparable brittleness constraints to PLA, and its fragility can be reduced by the addition of a specific plasticizer. Though PHA has higher barrier properties than other biomaterials, the disadvantages in manufacturing costs are higher and its recycling process is still under development [109,124]. While PET recycling methods are well known and commonly employed [6], there are few studies of the mechanical and chemical recycling of PHA due to the high production costs and low yields of the recycling process [125]. PHAs can then be manufactured and processed for use in many applications, including packaging, thermoplastic products, protective coatings, nonwoven textiles, resins, sheets and activity enhancers, to name a few. PHAs, in contrast to other biomaterials, have a lot of promise for applications such as packaging because of their superior thermal–mechanical and protective qualities [109].

### 3.6. PLA’s Traditional Sources

PLA is obtained from lactic acid precursors, which are produced from renewable sources such as sugar feedstock, straw maize, corn and food or agricultural waste products via fermentation [31,126].

Nowadays, fermentation represents the most common way to produce lactic acid, particularly if pure optical isomers are needed.

To obtain lactic acid, three main steps are required:

- In the first one, mono- and disaccharides are produced by the hydrolysis of carbohydrate sources.
- Then, lactic acid is attained by the fermentation of saccharides through lactic acid bacteria (LAB).
- Finally, the purity of lactic acid is obtained using further purification processes.

**Figure 5:**
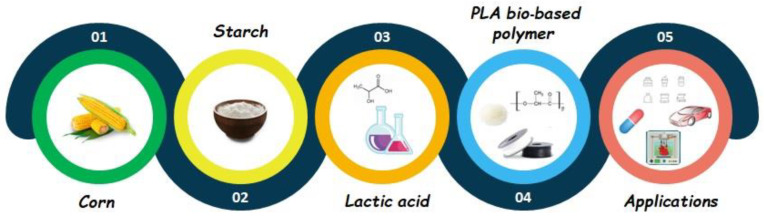
PLA from feedstocks to the final product.

The main sources of starch which can be used in the first step are as follows: corn (maize), straw, tapioca (cassava), potatoes and other raw materials which, after hydrolysis, are transformed into mono- and disaccharides (Figure 5). The hydrolysis of starch was first carried out by using chemicals, but nowadays enzymatic methods are preferred. On the other hand, only few bacteria are suitable for fermentation, because maltose results in the key product of enzymatic hydrolysis.

Furthermore, sucrose-derived raw materials can be used for the fermentation of lactic acid. Conversely, lactose has a limited use because of the low quantities which can be found in readily available whey, and it also requires a high purification of whey [127,128].

Afterwards, to produce lactic acid, different families of bacteria (LAB) can be used (e.g., *Lactobacillus*, *Streptococcus* and *Pediococcus*), which are characterized by high productivity in very narrow pH ranges [114] and allow the producing not only of lactic acid, but also of other organic acids during fermentation. Therefore, in order to focalize the production to lactic acid, decreasing as much as possible the number of other byproducts, the pH is kept between 5.5 and 6.5, thanks to particular bases such as hydroxides or carbonates.

To select the right raw material for the fermentation, some parameters must be considered, such as the availability, the price and, above all, the purity, which influence the field of application of PLA. Mono- and disaccharide traditionally employed mainly derive from the conversion of different substrates and are:

− Glucose and glucose syrups deriving from the conversion of starch with enzymes such as glucoamylases;
− Maltose, derived from the starch enzymatic conversion with amylases of malt;
− Sucrose, obtained as a byproduct or intermediate of cane sugar;
− Lactose, derived from mil whey, a natural substrate of several lactic acid bacteria.

### 3.7. PLA from Wood Wastes Sources

Lactic acid, traditionally used in several fields (chemical, cosmetic, food industries and pharmaceutical), is a hydroxycarboxylic acid characterized by two optical isomers [129,130,131].

Recently, the use of optically pure lactic acid (l- or d-isomer) was studied as a building block for the polylactic acid (PLA: PLLA and PDLA). As already mentioned, PLA is one of the most eco-friendly biomaterials and can be used as a green alternative to traditional fossil polymers [132,133]. Nevertheless, the traditional ways to produce PLA cause the subtraction of important sources from the food chain; therefore, many efforts are currently made to obtain PLA from scraps, thus producing a biopolymer of the third generation.

An innovative way to optimize PLA mechanical properties, using optically pure lactic acid isomers, involves the mixture of pure PLLA and PDLA, thus obtaining Sc-PLA, a stable stereo-complex with good mechanical properties, higher hydrolysis resistance compared to the use of a single enantiomer [134], a melting point ~50 °C higher than the pure materials and higher biodegradability [135].

**Figure 6:**
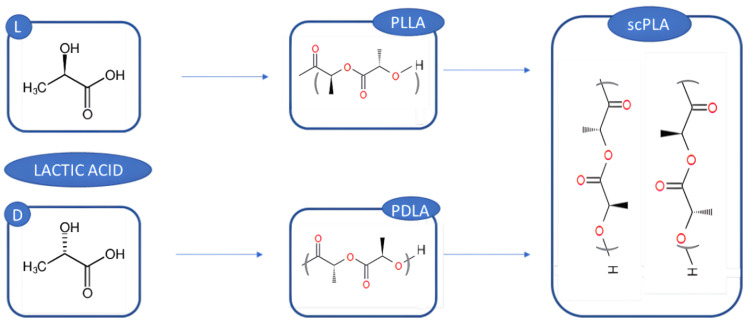
Synthesis pathway of PLA.

The pure isomers can be obtained through microbial fermentation [136,137], although there are many issues which hinder the scale-up of the production from laboratory to industries, such as the high cost, the raw materials and the nutrient sources [136,138]. In order to solve these problems, second-generation feedstocks, which involve lignocellulosic biomass from agro-industrial or forest sources, are nowadays studied as inexpensive renewable sources for lactic acid production and the consequent microbial fermentation to produce PLA isomers [139,140] (Figure 6).

Lignocellulosic feedstocks, although seemingly a promising way to replace traditional sources for PLA production, are characterized by a complex structure; therefore, their conversion in optically pure lactic acid is still a challenge [141]. Furthermore, all the feedstocks must be pretreated in order to remove the lignin, thus allowing the enzymes to access the cellulose. Furthermore, inhibitions of the enzymatic catalysis can occur due to the pretreatment of the material; the inhibition mainly consists of the slowdown of both cellulose hydrolysis of lignocellulosic biomass and of the microbial growth [142].

Another drawback which occurs with the use of lignocellulosic scraps is that they are composed of a heterogeneous mixture of sugars, which cannot be easily used at the same time.

The main bacteria often used to produce lactic acid are lactic acid bacteria (LAB). Nevertheless, their use is still not widespread due to many factors, such as the requirement of specific nutrients, the low resistance to acid and the difficulties with co-utilizing glucose and xylose [143]. Recent improvements in bacterial D-lactic acid involve a genetic modification [144,145]. Some of the recent advanced processes, aiming to increase D-lactic production, include fed-batch fermentation, continuous fermentation with cell recycling and integrated membrane fermentation [146].

The industrial and commercial employment of lignocellulose to produce lactic acid is still an issue. In fact, various processing steps are necessary to convert the lignocellulosic biomass to monomeric sugars that are then fermented to obtain lactic acid. The conventional processes used to produce lactic acid from wood biomass involve four main steps: First, the lignocellulosic raw material needs to be pretreated, to break the structure of the biomass; after pretreatment, enzymatic hydrolysis occurs, thus obtaining fermentative sugars through hydrolytic enzymes. The third step consists of fermentation, which allows the metabolization of sugars to lactic acid, usually via LAB. Finally, in the fourth step, the lactic acid is collected and purified. It is worth highlighting the important role of the pretreatment of lignocellulosic biomass, since the native lignocellulose has a low enzymatic susceptibility due to the association of cellulose and hemicellulose with lignin [147,148]; therefore, the efficiency of the pretreatment plays a key role for the subsequent saccharification via hydrolytic enzymes. Finally, if the pretreatment is too strong, toxic materials can be released with a consequent inhibition of the microbial metabolism and growth [149].

## 4. Main Properties of Bio-Based Polymers and Biocomposites

**Figure 7:**
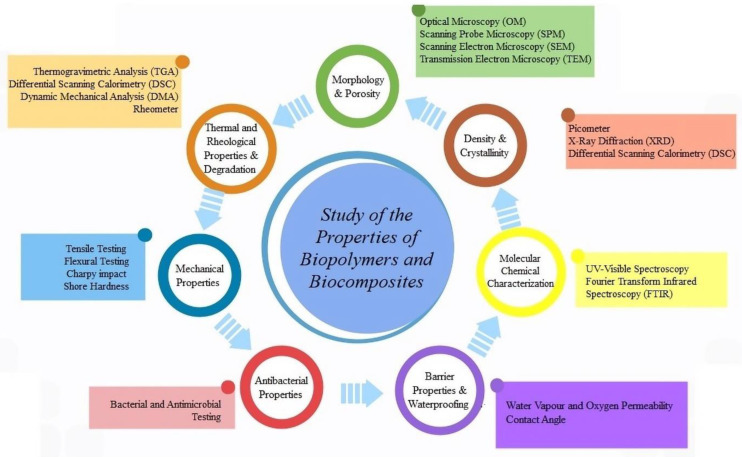
Some of the characterization methods commonly used to study the main properties of biopolymers and biocomposites.

Generally, the most investigated features for newly developed biofilms and biocomposites are morphological, thermal, mechanical, rheological, water absorption and antibacterial properties [40,150,151,152,153]. Their characterization involves the use of specific analytical techniques, some of which are discussed in this section and shown in Figure 7. Specifically, microscopy (Scanning Probe Microscopy SPM, Scanning Electron Microscopy SEM and Transmission Electron Microscopy TEM) is utilized for the measurement of morphology and porosity, the picometer is useful for density measurement, X-ray Diffraction (XRD) allows one to determine crystallinity, spectroscopy (UV-Visible and Fourier Transform Infrared Spectroscopy FTIR) is employed for molecular chemical characterization, thermal analysis (Thermogravimetric Analysis TGA, Differential Scanning Calorimetry DSC and Dynamic Mechanical Analysis DMA) is employed for the measurement of thermal properties and the study of thermomechanical degradation, water and oxygen adsorption is considered for the evaluation of barrier properties and Tensile and Flexural Testing, the Charpy impact test and Shore Hardness are utilized for the measurements of mechanical properties, etc. [40,150,151,152,153,154,155]. Usually, the environmental impact and biodegradability of biopolymers and biocomposites are evaluated according to American Society for Testing and Materials (ASTM) standards [153].

The mechanical qualities of biopolymers include strength [15], ductility [156], deformability [157], stress [158] and durability [159]. Mechanical properties are usually detected using a dynamometer and by choosing the geometry, the load cell and the test speed according to the appropriate standard test method. Six replicates for each measurement are usually performed, in order to obtain statistically relevant results. Durability tests are carried out on a climate chamber, following the ageing procedure described in the standard test method. The degradation in the flexural modulus, in the strength of the composites and in the interlaminar strength are evaluated after the ageing.

The intrinsic qualities of a polymer are determined by its structure and/or chemical composition [160]. Density is a fundamental feature of polymers, varying between classes and constituents [160] and often measured using a pycnometer. In comparison to petroleum-derived polymers, most biodegradable polymers have higher densities.

Though crystallinity isn’t considered a self-reflective feature, it affects many other qualities. High-crystalline biopolymers are more resistant to dissolution than low-crystalline biopolymers. A single biopolymer’s crystallization degree affects its melting and glass transition temperatures [160], as well as affects solubility; with greater compactness of the structure, there is a lower chance of dissolution [160]. In order to evaluate the crystallinity of the material, both X-Ray diffraction and Differential Scanning Calorimetry are used.

Scientific studies show that the tensile strength, and thus the crystallinity and solubility of biopolymers, can be increased by inserting different kinds of fillers into the polymer matrix, obtaining biocomposites [161]. For example, both PLA and polyolefin-based composites containing wood fillers have similar mechanical properties [161]. Generally, the addition of wood filler to the polymer matrix has different effects on tensile strength. In fact, the measured values arise from the wood species used as fillers and the amount added, the composites’ processing methods and the quality of the PLA [161,162,163]; an additional cause of the reduced mechanical properties of the polymer composites with wood filler is also due to the poor adhesion at the interface between the filler and polymer matrix [154]. This phenomenon is especially evident from high-magnification images obtained using scanning electron microscopy (SEM) on both biofilms and biocomposites and/or biofilaments for 3D printing. For example, some authors report that the transparent films produced have pores and cracks that facilitate the passage of water vapor and gases [150]. Alternatively, other works highlight in SEM images of composites in general, or composite filaments for 3D printing, an irregular, rough, outer surface with several points of discontinuity due to the addition of wood filler, which is often non-homogeneously distributed in the polymer matrix, with the formation of particle aggregates [154,164]. Furthermore, the presence of voids and poor interfacial adhesion between layers (observable in composite sections and often on 3D prints as defects between layers) are attributed to the different polarity between the biopolymer (which has a non-polar surface) and the wood fillers (which have a polar surface) [154,164]. Instead, SEM results often indicate correct dispersion of the filler in the polymer matrix and an improved hydrophobic nature compared to the neat polymer film [151]. Overall, the results depend on the physical and mechanical properties of the polymers and fillers, concentration of the parts, filler geometry, polarity, compatibility, addition of plasticizers and type [151,152,154,165,166]. However, most studies show that, overall, the stiffness of polymeric composites with wood fillers increases, as does the crystallinity compared to pure polymer, and scientific research has focused on improving structural properties, such as through the use of plasticizers [161]. XRD and DSC analyses are useful in order to evaluate the effect of the addition of a plasticizer to different kinds of biopolymers. As reported by Greco et al. [155], the mechanical properties of PLA can be tailored by the addition of different green plasticizers. The plasticization of PLA can be carried out via the extrusion of the polymer with a specific amount of different bio-based plasticizers (e.g., neat cardanol, epoxidized cardanol acetate (ECA) and poly(ethylene glycol) (PEG 400)). Results evidenced the plasticization effectiveness of the different additives in terms of the reduction in glass transition temperature: compared to neat PLA, which showed a Tg of 60 °C, the addition of PEG 400, cardanol and epoxidized cardanol involved a decrease in the Tg to 17.5 °C, 16.9 °C and 22.3 °C, respectively. On the other hand, the addition of the plasticizer can influence the degree of crystallinity of the polymer: faster crystallization and an increase in the degree of crystallinity was found for PLA plasticized by PEG, compared to PLA plasticized by cardanol derivatives. Furthermore, mechanical properties were influenced after plasticization, which involves a decrease in the stiffness of the polymer. In particular, as reported in Table 3, different results were obtained if the plasticized polymer was amorphous or semicrystalline; neat amorphous PLA had a Young modulus of 1740 MPa. When PLA was plasticized with cardanol derivatives and an amorphous structure was detected after plasticization, a good efficiency occurred; however, the thicker crystals formed during crystallization of PLA with PEG and cardanol derivatives led to a high increase in the stiffness of the material.

Xie et al. [167] studied the influence of plasticizing agents (glycerol and tributyl citrate, TBC) on the stability and water absorption of samples based on poplar wood flour and PLA for 3D printing. Two different weight concentrations were tested for each plasticizer agent, 2% and 4% wt. Good compatibility between filler and polymer, good interfacial adhesion and good mechanical properties were obtained using TBC at 4% wt. [167]. In contrast, Zhang et al. [168] developed high-yield esterified lignocellulose nanofibers (LCNFs) from lignocellulose (LCs) by swelling with a deep eutectic solvent lactic acid/choline chloride (LA/ChCl DES) (100 °C for 3 h), followed by mechanical colloidal grinding. LCNFs/PLA composites were obtained via direct mixing, and the morphological, structural and mechanical properties were studied. The authors showed a significant improvement in compatibility at the LCNF/PLA interface highlighting the potential of natural wood-derived nanofibers for making bio-based composites [168]. However, there is an impact of plasticizers on tensile strength up to a threshold value, because the tensile strength decreases with an increase in the amount of plasticizer, particularly in the case of films produced from starch [169]. Mechanical properties are lower for wood–PHA polymers, and even more so for starch. Young’s modulus, tensile strength and stiffness decrease for wood–PHA composites, while strain at break is higher [161,170]. Scientific interest in PHA polymers with wood is growing due precisely to their sustainability and interesting and modular mechanical properties [161,170]. For example, Mehrpouya et al. [171], in their work focused on 3D printing, report some examples where the combination of PHAs and natural wood-derived additives (i.e., fibers, lignin, fibrillated nanocellulose, cellulose nanocrystals, etc.) makes remarkable improvements to the strength and microstructural features of pure PHAs. Wu et al. [172] achieved 20% higher parameters of tensile strength at break (7%) and Young’s modulus (65 MPa) than pure PHAs by developing a wood–PHA composite (PHA-g-MA/TPF) with polyhydroxyalkanoate engaged with maleic anhydride (PHA-g-MA) and palm fiber treated with coupling agents (TPF) [172]. In addition, the use of wood–PHA composites also offers the possibility of reducing costs compared to pure PHA, while still using a sustainable material [170,172].

Thermoplastic starch (TPS) is the least-used material for the development of wood plastic composites (WPCs), compared to materials such as PLA and PHAs; it has poor mechanical and water barrier properties, and difficult processability [31,161]. Yet, wood filling to the polymer-based specimen results in improvements in tensile strength at break, elastic modulus and elongation at break, and these changes are even more pronounced than with other biodegradable polyesters, due to greater compatibility between the two materials (starch and wood) due to the hydrogen bonding of OH groups [161,173]. Zeng et al. [174] investigated the consequences of three additives (plasticizer, cross-linking agent and blowing agent) on the mechanical properties of a foamed composite made of starch, wood fibers and polyvinyl alcohol (PVA), using a predictive model. The work shows how using the appropriate amount of plasticizer (glycerin/NaOH) can reduce the crystallinity of starch and increase the compatibility between the different components and the tensile strength of the ultimate material (maximum range of 5.91–6.12 MPa) [174].

Dorigato et al. [175] improved the final mechanical properties of fully biodegradable composites laminated with starch and beech wood by impregnating them with poly(ethylene glycol) (PEG) and consolidating via hot pressing [175]. Alternatively, Harussani et al. [169] analyzed various quantities of two plasticizers (sorbitol and glycerol) in different concentrations by weight (30%, 45% and 60%) on cornstarch-based composites, obtaining better mechanical performance with the use of 30% sorbitol: increased tensile strength (13.61 MPa) by 46% and unchanged elastic modulus [169].

Unlike PLA and PHAs, research on starch-based bio-based composites has focused more on understanding the increase in mechanical performance due to different types of wood fillers, and not on increasing the adhesion of the interface and material compatibilities through the use of plasticizing agents [161,174]. For example, Curvelo et al. [176] measured a 100% increase in tensile strength and 50% increase in elastic modulus, compared to pure thermoplastic starch, after the addition of Eucalyptus urograndis pulp fibers to the composite in 16% wt. concentration [176].

Until today, the use of polymeric degradable matrices for the production of composites has also sometimes been reduced due to their poor moisture and gas barrier properties and limited stability [161]. Barrier, rheological and thermal properties are in fact also of great importance, and are closely related to the structural and mechanical properties already mentioned. For example, the glass transition temperature (T_g_) of different pure materials exhibits great differences (i.e., Starch 31–98 °C, PLA 45–60 °C, PHA −4–8 °C) [161], and the addition of wood fillers makes different changes to this and to the viscosity and processability of wood–bioplastic composites [154,169].

Polymer barrier qualities are strongly linked to their capability to allow the interchange molecules that have small size. The form, orientation and crystalline nature of the diffusing molecule, as well as the degree of polymerization and polymer chains, influence the barrier qualities. No material is totally resistant to ambient gasses, water vapor or to other generic natural compounds. In terms of ability of the biopolymer to permeate water or oxygen molecules, generically it is possible to identify three classes: permeable to both water vapor and oxygen; low ability to permeate water vapor but high barrier against oxygen; or contrastingly, less permeable to oxygen but very permeable to water vapor [161]. Among all bioplastics, starch-based composites usually exhibit higher hydrophobicity and low water resistance [31,161,174]. For this reason, several scientific studies have also been conducted to improve the barrier features of starch-based materials by modifying the structural properties through natural fillers of different origins. Curvelo et al. [176], in their starch-based composites developed by the addition of wood pulp (Eucalyptus urograndis at 16% wt.), also measured a significant improvement in water absorption properties. The new composites were conditioned by maintaining relative humidity values of 43 and 100%, and a temperature of 25 °C, and the water absorption values were found to be almost halved, unlike the values of neat starch [176]. Miranda et al. [177] studied changes in the structural and absorption properties of flexible thermoplastic films of corn starch, following the addition of cellulose nanocrystals (CNC) as reinforcement, and measured a significant improvement in the stability and barrier properties of pure starch [173]. Alternatively, Chen et al. [178] investigated the addition of nanoscale cellulose particles of different types (bamboo, cotton linter and sisal) to starch samples, using different content (0–10 wt%), and obtained better water vapor barrier properties with the use of bamboo nanocellulose, among others [178].

PLA and PHA-based composites, on the other hand, have higher intrinsic water resistance properties than starch [161]; however, the water barrier properties of biocomposites, to which a wood filler is added, decrease as the percentage content of wood particles/fibers increases and improvements are needed [178]. Da Silva et al. [178] studied the reaction of Struktol used as an adjuvant on the final features of wood–PHB biocomposites, showing a slight improvement in water absorption properties only for the sample containing 20% of wood filler [178]. Wu et al. [172] showed lower water absorption of the developed wood–PHA composite (PHA-g-MA/TPF) with polyhydroxyalkanoate grafted with maleic anhydride (PHA-g-MA) and palm fiber treated with coupling agents (TPF), compared to the corresponding PHA/PF composite [172]. In addition, Song et al. [179] focused their work on increasing the water vapor barrier characteristics of PLA films by using nano-cellulose fibers (NCFs) modified by adding hydrophobic molecules onto the NCFs to increase the compatibility between NCFs and PLA during mixing. Paper water vapor transmission rate (WVTR) tests were conducted following different operating parameters and with different weight coatings showed that the coating of NCF/PLA innovative samples reduced the WVTR (up to 34 g/m^2^/d) [179].

Some important properties of the main types of bio-based wood composites are reported in Table 4.

## 5. Market Scenarios and Applications of Biopolymers and Biocomposites

**Figure 8:**
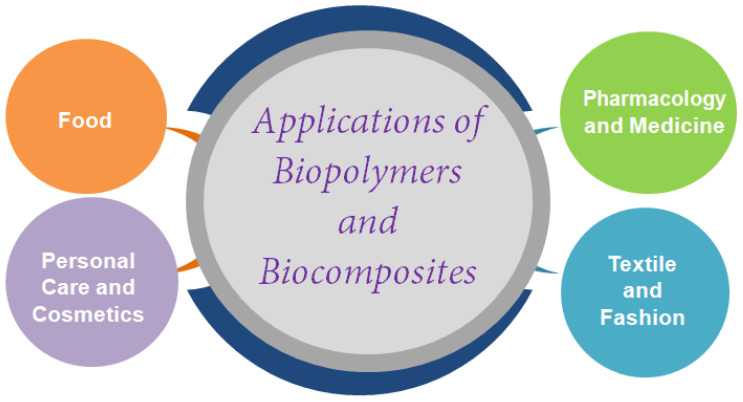
Main application of biopolymers and biocomposites.

The global bioplastics market is growing significantly due to an increased focus of manufacturers and users on the use of sustainable products, social and economic factors and the more restrictive legislation implemented in recent years [192]. For example, the United Kingdom (UK) introduced a new tax from April 2022 on plastic packaging (called “Plastic Packaging Tax, PPT”), locally produced or imported, containing less than 30% reused plastic by weight, to increase the use of biopolymers, recycling and sustainable development. Aside from this, the tax in Italy has been postponed to the year 2023 [193]. Overall, estimates report a likely increase in bioplastics production from the current USD 9.2 billion to USD billion by the year 2026 [194]. Among different geographic areas, Asia is the largest production center (producing 46% compared to total bio-based polymers produced worldwide), followed by Europe (26%) and North (17%) and South America (10%) [27,194]. Europe is the geographic area where research activities on bioplastic development are most concentrated, and biopolymers are especially used in food packaging (60%) followed by other industries such as agriculture (13%), consumer goods (9%), coatings and adhesives (9%), textiles (5%), automotive (1%) and all others (3%) [27,192]. Several elements influence the market scenarios of bio-based polymers, and consequently also the application areas: the availability of raw materials and renewable sources, the ease of scalability and the production process, the biodegradability features (i.e., durability, degradation conditions and end-of-life treatment) and costs [27]. By cost it is not only meant that which is associated with the recovery of the raw material, but also that of its processing which must be suitable for market demands so that users prefer biopolymers to traditional polymers of petrochemical origin, which usually possess good mechanical and structural properties and often low costs [192,195]. Cost becomes a significant factor, especially in areas known for the development of single-use plastics for food packaging or low performance. Usually, bioplastics are more expensive than petroleum-based plastics. In addition, bioplastics also often have a higher density, causing further cost increases. However, there are exceptions when prices are compared at the product level; redesign and specific material properties can result in raw material savings, for example, due to the higher stiffness of PLA compared to PS, rigid PLA products can be reduced in thickness or the use of additive techniques, which involve adding natural or recycled fillers to the polymer matrix, can further reduce the initial cost of the biopolymer [12,154,196,197]. For example, in the European market, Nylon 6 (virgin polymer, PA6) costs around EUR 2.5–4.0/kg, while low-density polyethylene (LDPE) costs EUR 1.1–2.7/kg and PET costs around EUR 1.60–5.0/kg [193,198]. The price of petroleum-derived plastics is likely to rise further, due to rising raw material and energy costs in recent months. However, the cost per EUR of bioplastics has decreased significantly in recent years; for example, PLA, which had a cost of EUR 7/kg in the 1990s has dropped to about EUR 1.6–2.5/kg in 2022 [198]. Rising oil prices have thus made the cost of bio-based plastics similar to that of thermoplastics of synthetic derivation; moreover, recent progress in the development of biopolymers has caused an increase in demand in different industrial sectors, such as food [110,199,200,201], pharmaceutical and medical [45,202,203,204,205], personal care [206,207], cosmetics [206,208] and textile and fashion [12,209,210] (Figure 8).

### 5.1. Biopolymers in Food Industry

Generally, several applications of biopolymers sintered and extracted from animals, plants or marine organisms are known in the literature in the food field. Among these, extracts from plant species, such as cellulose, lignin, polyphenols and essential oils have been extensively used for different aims [211]. For example, they are used for the fabrication of edible films and coatings [212]; these constitute a thin layer of soluble bioactive compounds that is applied to the surface of foods or between layers for different purposes such as extending food shelf life, improving quality or acting as a barrier for oxygen, water and solutes [153,213]. Specifically, these compounds are extracted using conventional or innovative methods for the development of edible films and coatings [214]. Improved mechanical and barrier properties of these films have been achieved through the use of composites (derived by combining multiple biopolymers and layers or by adding fibers and fillers to the biopolymer, such as laponite, montmorillonite, sepiolite, palygorskite, etc.) or biopolymer nanocomposites (i.e., by the addition of nano SiO_2_-x, nano ZnO, nano oxide and nano TiO_2_, etc., to the polymer) [214]. Typically, these biopolymer-based coatings, in addition to being biodegradable and nontoxic, may have a natural microbial action; others can serve as a carrier for antioxidant or antimicrobial biopolymers [215]. In fact, better antibacterial, antifungal or antioxidant properties of edible films have been achieved by incorporating active compounds (i.e., antimicrobials, antioxidants, dyes, flavors and nutraceuticals) into filmogenic solutions [216]. For example, organic acids and essential oils (EOs), which have intrinsic antimicrobial and antioxidant properties, have been added to biopolymers derived from cellulose and derivates [217], i.e., thyme, clove, rosemary, oregano, cinnamon and tea oils [211]. Polyphenols (i.e., phenolic acids, flavonoids and proanthocyanidins) are other important active compounds added to polymeric edible films for their antioxidant, aromatic and antimicrobial properties [211]. Scientific studies have shown that nanomaterials can also be used to control the release of bioactive agents incorporated into edible packaging, improving its durability [212]. Several reviews have been written in recent years highlighting the progress made by research in the development of new biopolymer-based edible films/coatings [212,214,216]. For example, Das et al. [214] emphasized in their work the use of no thermal techniques (such as cold plasma, ultrasound, UV irradiation, high-pressure homogenization) and the addition of nanomaterials (nanoparticles of silver, zinc oxide, titanium dioxide, montmorillonite) to improve the structural (color, thickness, intermolecular bonds, particle size), water and oxygen barrier properties and mechanical features of edible films. Kumar et al. [212] reported the different natural materials used for either pure or composites and, for each, reported the preparation techniques of edible films/coatings and the antimicrobial, antioxidant, physical and sensory properties, also highlighting any critical issues, recent applications and commercial products. In the last few years, the growth of concern for the environment has led to a preference for the use of agricultural and industrial scraps for the development of edible films/coatings that provide a feasible alternative to the use of plastics or bio-based materials of natural origin or from biomass, in favor of sustainable development and the circular economy [212,216,218,219].

Biopolymers are mainly used in food packaging [110,153,220]. The causes mainly can be attributed to the problems that have emerged from the disposal of traditional petroleum-derived materials and the mandatory regulations now existing and increasingly developed in recent years in food packaging, as well as the significant increase in the cost of petroleum products, due to various reasons [213]. Food packaging is supposed to extend the conservation life of all sorts of foods by storing and protecting them from oxidative and microbiological degradation. Next-generation food packaging must have certain durability and good mechanical and barrier properties, as well as aesthetic functions related to marketing [219]. Among the natural polymers most commonly used for food packaging, cellulose and derivates are usually treated, processed, melted and dried. Chitosan, for example, is an antibacterial biopolymer. The encapsulation of chitosan has an important place in obtaining food and packaging with a long shelf life, as demonstrated in a recent study by Baysal et al. [221]. Other biopolymers used for food packaging are PLA, PHA, PBAT or blends of biopolymers, such as a starch-based blend with PLA, PHB, PHAs, PVAs, PCL, PVOH, etc. [149]. Torres-Giner et al. [222], after a classification of polymers used extensively in food packaging (divided into petroleum materials, and into non-biodegradable and biodegradable products), focused the paper on materials such as PLA, PHAs, PBAT, PBS, bio-based PET, cellulose and derivatives of different origins. Similarly, as for edible films, for food packaging the best mechanical, physical and antimicrobial barrier properties have been achieved through the use of multilayers [223] and by adding active ingredients (usually in the form of a sachet or covering on the packaging material embedded in the surface of the material itself) [211] or even nanometer fillers to biopolymers (nanofillers) [224]. In the former case, traditional multilayer packaging comprising polymeric layers (i.e., PE, PET, HDPE, PP, EVO, EVOH, PA, etc.) and inorganic layers (i.e., Al, SiOx, etc.) [223] is being replaced by biocompostable packaging called “active” packaging, which has a reduced environmental impact. Wang et al. [200] report several advanced examples of biodegradable active multilayer packaging composed of sandwich-like substances. These are based on polymer matrices of different natures (among them, methylcellulose has often been used) and active components, such as polyphenols, potassium sorbate, lysozymes, etc. [200]. The authors in the paper examine different production techniques, focusing on a mathematical pattern for release control of the active component of the packaging [200]. In the second case, nanofillers (classified into nanoplatelets, nanofibers and nanoparticles), having nanometric structures and antibacterial properties, are added to biodegradable and environmentally friendly polymers in so-called “smart” packaging (which allows the freshness of the food to be checked in real time), which has recently been added to “active” packaging (in which the added substances protect the food from UV rays, oxygen and microbes interacting directly with the food) on the market. The addition of nanofillers to green polymers produces a number of benefits, such as reduced risk of spreading pathogens, improved food quality, reduced material waste and sustainability [224,225]. Among these, the most widely used nanofillers are antibacterial nanoparticles (such as Ag, ZnO, Cu/CuO, TiO_2_, Fe_2_O_3_, Fe_3_O_4_ and MgO), mesoporous particles, graphene and carbon dots, added in green polymers [224,225]. However, recent research highlights the importance of prior evaluation of the safety of metal oxide nanoparticle additives (through migration testing), and biocompatibility with polymers to minimize the risk of toxicity [225].

The scientific literature has developed several studies on natural fiber-based biopolymers too. Different lignocellulosic fibers, such as wheat straw, linen fibers, jute, coconut, kenaf and olive pomace, have been investigated for their usefulness as fillers [110]. Even if there is an increasing awareness of environmentally friendly packaging, it is necessary to use bio-based and sustainable packaging solutions. Next-generation packaging (often called 4G) combines all these properties with environmental friendliness, through the use of innovative materials [226]. The commercial and technological potential of industrial by-products for the production of next-generation (active and smart) food packaging to support zero waste activities has been known for some years now. For example, Bhat et al. [227] used lignin derived from oil palm black liquor scrap added to sago palm (*Metroxylon sagu*) films for the development of food packaging. After extraction and solubilization in DMSO, the lignin was added in several percentages (from 1 to 5% *v*/*w*) to the starting solution to form the packaging films. The authors in their work demonstrated an increase in the mechanical properties, resistance to thermal sealing, water vapor permeability, solubility and thermal stability of the films obtained using lignin produced from waste [227]. Sánchez-Safont et al. [110] tested the use of local lignocellulosic scraps (rice husk, almond shell and sea grass) as additives for the development of PHB/fiber composites for use in food packaging. Improved mechanical and permeability properties of the composites were obtained, as well as improved thermoforming ability of the films. Tumwesigye et al. [228] used bitter cassava waste to develop a low-cost food packaging film, turning environmental waste into a sustainable resource. Specifically, two different transparent films were produced and tested by the authors, from intact and decorticated bitter cassava; among these, the former were shown to have the best mechanical qualities and structural and higher thermal stability, while leading to a higher yield with a 16% reduction in waste [228].

### 5.2. Biopolymers in Pharmacology and Medicine

Biopolymers and their composites are also used in the pharmacological and medical fields due to their biodegradability, cost-effectiveness, wide availability, processability and especially biocompatibility with human organs and tissues [153,205,229]. For all of these qualities, they are used, for example, as materials for transporting pharmaceutical molecules and substances (such as enzymes, antibiotics and antineoplastic drugs, etc.), in ocular, dental, nasal and other systems [153,229,230]. Biopolymers from different sources and that have pharmaceutically active ingredients (capable of influencing the drug delivery process), are used for the production of Drug Delivery Systems (DDS), usually in the form of microcapsules, microspheres, nanospheres, hydrogels, nanogels and liposomes [153,229]. In this area, polysaccharides have been exploited for years mainly for their properties that can form linkages with proteins and lipids. Among the polysaccharides, cellulose (together with starch) is the most widely used in pharmacology and medicine, in general [203,231]. The use of cellulose (and its derivatives) for the development of DDS has expanded due to its exceptional properties, such as its ability to absorb and retain water, its biocompostability and its structural characteristics that allow the loading of specific molecules [203]; in addition, the possibility of producing nanocellulose from wood pulp, along with the use of advanced technologies (such as 3D printing), has opened up an opportunity for the development of innovative materials for pharmacological applications in recent years [231]. For example, Yu et al. [231] used ethylcellulose and hydroxypropyl methylcellulose for the development of a drug delivery device, prepared automatically using 3D printing, that was capable of providing a linear release profile of acetaminophen. Specifically, hydrophobic ethyl cellulose delays the initial rapid release of the drug, while hydroxypropyl methylcellulose swells into a gel after contact with the dissolution medium, releasing the drug for an extended period [231]. Biopolymers are also used in the medical field for the production of hydrogels and nanogels, materials suitable for wound healing [203,232,233]. Several hydrogels and biopolymer-based formulations have emerged from the addition of therapeutic and bioactive agents such as antimicrobials, growth factors, antioxidants, antiseptics, etc., that facilitate the skin healing process [203,232,233]. Wound-healing materials use hydrocolloids such as foam, gel or spray [153,203,232]. Many researchers have invented several methods for the development of cellulose, hemicellulose and lignin from agricultural wastes, such as from sugar beet, cashew nuts, sago waste, waste from the cotton ginning industry, etc., using a variety of techniques [234,235]. Among them, Cui et al. [236] tested aqueous-based hydrogels from cellulose derived from industrial durian rind waste, fortified with glycerol to obtain organohydrogels, which proved to be suitable for antimicrobial wound dressing, even under extreme thermal conditions (for example, −30 °C). In their work, Amores-Monge et al. [237] investigated the opportunity to build a high-profit market that focuses on the production of products (i.e., cellulose, hemicellulose, lignin and enzymes) with biomedical applications from waste obtained from pineapple (*Bromeliaceae family*); among them, the proteolytic enzyme bromelain was found to have an essential application in skin reconstitution [237].

Properties such as biocompatibility, biodegradation and noncytotoxicity also make biopolymers excellent candidates for use in implantable medical materials and scaffolds [153,238,239]. Implantable medical devices are predominantly used to simulate and replace a human structure that has been damaged, or to support normal body function or control trunk posture [239,240]; scaffolds, on the other hand, are used to facilitate hard and soft tissue regeneration in tissue engineering [153,238,241,242]. Bones, heart, eyes, ears, knees, hips, etc. constitute the anatomical parts that are undergoing integration and replacement with polymeric medical implants the most [153]. Teeth, bones and the cartilage of humans, in contrast, are the human parts most concerned with the application of scaffolds [243,244]. Today, traditional materials such as metals and ceramics have been almost completely replaced by biopolymers, due to the immunological rejection by the body that they can cause; biopolymers, on the other hand, exhibit biocompatibility, good degradability, renewability, anti-toxicity and antibacteriality throughout their life cycle [202,238,239]. For the fabrication of medical devices, the materials usually used are polylactic acid (PLA), polyglycolic acid (PGA), poly(lactic-co-glycolic acid) (PGLA) and polycaprolactone (PCL) [153,243], also known as nanocomposites (nanotubes, nanoparticles and nanofibers) [12]. These polymers have also been combined to produce implants. For example, copolymers of PLA and PGA have often been used in place of their respective homopolymers in orthopedic applications (e.g., for the creation of plates or screws for the treatment of fractures and the filling of bone defects) [153]. Alternatively, PLA and PET have been combined to produce prostheses for vascular surgery [153]. The development of Additive Manufacturing (AM) techniques has also affected the biomedical and tissue engineering fields: 3D printing of tissues, organs and body parts using biopolymer nanocomposites has been made possible by the spread of some easy and low-cost 3D-printing techniques, which also have the advantage of printing complex geometries [12,202,240]. For example, S. Bartlett [245] reports on the 3D printing of a bioresorbable tracheal splint that was successfully implanted in the patient and was produced by combining TC images of the airway with the 3D printer. Gross et al. [246] report that 3D printing has also been used for the reproduction of anatomic parts needed for the preliminary study of surgical procedures, for example, to create a calcified aorta with 3D printing for the study of plaque removal surgery, to optimize the removal of bony outgrowths on a shoulder and for the study of drug delivery into the lungs of a premature infant [246]. The fabrication of scaffolds for bone tissue regeneration requires good biomimetic and bioactive properties; therefore, micro- or nanoparticles, comparable to the natural mineral components of bone, are often added to traditional polymers, such as tricalcium phosphate (TP), hydroxyapatite (HA), calcium phosphate cements (CPC), monetite or brushite [246]. For example, in their work, Corcione et al. (2017, 2018, 2019) [242,243,247] explored the possibility of using FFF printing to develop an osteogenic bone graft based on hydroxyapatite (HA) and polylactic acid (PLA). PLA, PGA, PCL, etc. have also been used together with other biopolymers, such as hyaluronic acid, cellulose, collagen, gelatin, elastin and fibroin, for the synthesis of tissues such as adipose, ligaments, blood vessels, liver, cartilage, pancreas, spinal cord and bone regeneration [153,244]. For example, collagen was often mixed with PLA, PGA, PCL, etc., to improve wettability and interaction with biological substrates [248,249].

### 5.3. Biopolymers in Personal Care and Cosmetics

Biopolymers are also used for the production of personal care products and cosmetics [207,250]. The first group includes sanitary napkins, panty liners, feminine hygiene pads, baby diapers and adult incontinence items, which are aimed at improving peoples’ lifestyles [239]. These are products that possess multiple layers, each with specific functions, and are composed of different types of synthetic or natural raw materials [239]. So-called “Superabsorbent polymers” (SAPs) constitute the main absorbent component of the layered structure of a personal care product (such as diapers or sanitary napkins for babies and adults). SAPs are a group of cross-linked hydrophilic polymers that are suitable for absorbing aqueous solutions, such as blood and urine, in a short time while keeping the skin dry and limiting infection or irritation [239]. For example, the most well-known SAPs used in personal care are polysaccharide SAPs (such as cellulose, hemicelluloses, bamboo, etc.), in addition to protein SAPs [250]. The demand for hygiene and personal care products will increase in years to come; conversely, environmental concerns over the use of products that are not fully biodegradable are growing, and there is an increasing need to instigate research activities aimed at producing non-toxic and eco-friendly products. Vivicot (Sanicot s.r.l., Prato, Italy) was the first line of compostable pads marketed in Italy (2011), made of pure organic cotton and certified by Certiquality [251]. Subsequently, the company Intimaluna (Borgo San Giovanni, Lodi, Italy) marketed fully compostable “Ecoluna line” feminine hygiene pads made of mater-bi and 100 percent organic cotton, menstrual cups and other washable feminine products [252]. Also in this area, scientific research is focusing on the recycling of raw materials for the production of new materials. For example, Lacoste et al. [253] produced a bio-based superabsorbent (bio-SAP) polymer for nappies from recycled cellulose. The authors demonstrated that they can recover and reuse waste packaging cellulose through a chlorine-free process, which transforms it into carboxymethylcellulose (CMC) that is cross-linked with citric acid afterwards [249]. In recent years, biopolymers have also been used in cosmetics due to their low cost, durability, versatility and biodegradability, and especially after the discovery of the presence of microplastics in aquatic ecosystems released from facial masks and scrubs, mascaras and lipsticks, shampoo, etc. [254].

Cellulose and derivates, polyhydroxyalkanoates, etc., are used in cosmetics for different purposes: for nanoparticle preparation or fragrance delivery, hair care, skin care and make-up [207]. Generally, biopolymers such as collagen, keratin and chitin are mostly used in this sector. For example, the major application of collagen hydrogels in cosmetics is to act as fillers for wrinkles and correctors of other skin defects [206]. Chitosan, on the other hand, is used more for hair care, and is included in shampoos, hair dyes, styling lotions, hair sprays and gels, or it is included in oral hygiene products, for the purpose of preventing tooth and gum disease [206]. Keratin, on the other hand, is mainly used for hair conditioning [206]. However, these substances are often used in combination with other polymers or biopolymers (such as cellulose and hydroxyethyl cellulose) or collagen hydrogel can be cross-linked with starch dialdehyde, tannic acid, squaric acid, PEG and other substances [206]. Examples of cosmetic actives derived from fish, meat, dairy and agro-industrial waste exist in the literature; products obtained from waste are a viable alternative to the usual plant extracts commonly used in cosmetic formulations, as they are effective, inexpensive and biosustainable [208,255]. In the area of industrial-waste-derived bipolymers, Meyabadi et al. [256] studied the reuse of waste cotton fibers and their conversion to cellulose powder for various applications, including cosmetics. The authors showed that spherical cellulose nanoparticles (less than 100 nm), produced through enzymatic hydrolysis followed by ultrasonic treatment, do not undergo significant changes in structure and major properties, providing a viable sustainable alternative [256]. In their work, Bongao et al. [257] highlighted the potential of micro- and nanocellulose extracted using conventional methods and synthesized from Pili pulp waste to replace the mineral ingredients used in cosmetics. Innovative research in the field of green chemistry and sustainable production now involves many companies. For example, the company Anomera (Montreal, Canada) has been awarded a 1.7 million grant to carry out in its research labs research into the replacement of environmentally harmful plastic microbeads with biodegradable, environmentally friendly, high-performance ingredients for cosmetics and skin care, made with cellulose derived from wood waste from the paper industry [258].

### 5.4. Biopolymers in Textile and Fashion

Biodegradable polymers also support the textile and fashion industry, by reducing raw material processing energy, materials and costs of sourcing, production and disposal [209]. In fact, the textile industry is one of the world’s most contaminating sectors, after petroleum; the greatest environmental damage comes from the production, processing and dyeing of the textiles [210]. This sector therefore needs alternative raw materials more than others; biopolymers are a responsible choice. Bio-based textiles, which must contain at least 20% renewable carbon, include natural materials and natural, synthetic or regenerated fibers [209,210]. Natural biopolymers are produced from polysaccharides (i.e., cellulose, lignin, etc.), as well as proteins and lipids of plant or animal origin [30]. In fact, natural fibers also come from plant sources (i.e., hemp, wool, cotton, etc.) [30,209,210]. Of these, cotton (together with silk and wool) is the most widely used in clothing production, as it meets aesthetic and wearability standards [30]. Synthetic and regenerated fibers used in textiles come from bacterial activities (such as polyhydroxyalkanoates, PHA) and from the synthesis of natural raw materials (e.g., polylactides, polyglycols, polycaprolactones, etc.) [30]. In recent years, the need for sustainable production has shifted the textile industry’s attention not only to materials such as organic cotton (grown without the use of pesticides, fertilizers or other chemical products), but also to the production of synthetic biodegradable textile fibers, referred to as “biodegradable nonwovens.” Among them, the biopolymers that find the most applications used in fiber spinning in the modern biodegradable textile industry are in fact polylactic acid (PLA), butyric acid (PHB), valeric acid (PHV), caprolactone (PCL), etc. [12,209].

Other examples of biodegradable nonwovens include those made of natural cellulosic fiber, cotton (cotton/cellulose or biodegradable cotton/co-polyester), the biodegradable nonwovens mentioned above and laminates (composites in which a layer includes a nonwoven fabric) [30]. In fact, composite materials derived from the addition of natural source fibers (such as hemp, flax, cellulose acetate, jute, pineapple, kenaf and many others) to synthetic biopolymers are often used [30]. For example, Gabryś et al. [259] transformed viscous, commercial, nonwoven fabrics by adding PLA (in addition to potassium nitrate, KNO_3_) to impart fertilizing properties to fabrics used in modern agricultural mulching. Many technologies are being developed to manufacture biosynthetic fibers from biomass and waste materials derived from agriculture, forestry and even food [30,201,209,260,261]. Several examples of biodegradable synthetic fibers are already commercially available, such as the biodegradable PLA thermoplastic Ingeo (company NatureWorks LLC, Blair, NE, USA) or Modal biofilters and Tencel/Lyocell products produced from beech and eucalyptus wood pulp (Lenzing Aktiengesellschaft, Lenzing, Austria) [209]. Commercial biodegradable products are often enriched with innovative antimicrobial agents to produce workwear, home wear, sportswear, etc. [209]. Early instances of biosynthetics using novel feedstocks such as algae, fungi, enzymes and bacteria are also available [30]. For example, the use of bacterial cellulose (produced by microorganisms) is growing in the textile sector, compared to the traditional use of plant cellulose, because it is sustainable, is biodegradable, does not pollute and can also be dyed, resulting in an attractive textile surface that meets current market research [210]. In addition, Patti et al. [30] report on several bio-based and sustainable textiles produced by large known companies from microbes, algae and bacteria for the production of jackets, shoes and other garments. The search for new biodegradable materials and the continuing evolution of traditional textile production methods, which usually involve the use of chemicals, have also led to the emergence of 3D-printing techniques [12]. Additive Manufacturing (AM) techniques enable the development of innovative and sustainable models for the textile industry and are being employed recently by major brands to shift the production of shoes, clothing, jewelry and other accessories to environmentally friendly and green materials [12,30]. Companies using 3D-printing techniques and biopolymers such as PLA and softened PLA (along with other materials such as, e.g., Ninjaflex, BendLay, TPE [262,263]), have resulted in nonwoven fabrics with improved morphological and structural properties compared to traditional polymers. For example, Loh et al. [264] developed and studied three different polymer composites for the textile world morphologically and mechanically, using a different combination of PLA, nylon and polyesters, and direct extrusion of the materials.

Table 5 reports the main properties and applications of bio-based polymers and biocomposites.

## 6. Conclusions

During the last decade, the production of bioplastics has mainly increased with the intention of decreasing the harmful effects of synthetic polymers on the environment. This study summarizes the current developments in the definition, classification, production, properties and applications of bio-based materials, particularly focusing on wood-waste derivates. The third-generation feedstock, obtained from food scraps, algae biomass and industrial or municipal waste, is the most promising category. It represents an innovative solution to the questions related to the consumption of sources from the food chain, according to the circular economy approach. Among the numerous varieties of organic wastes, agro-industrial and forestry wastes, which are generated in massive quantities each year, represent an unjustifiable environmental and economic scenario. The production of the main classes of biopolymers, starting from wood scraps, was reported in this work. Although lab-scale experiments showed promising ways to produce biopolymers from lignocellulosic wastes, the industrial production is still not sufficiently profitable, due to the high cost of the processes. Nevertheless, thanks to the relevant benefits obtained by the use of wood scraps, several studies regarding the development of genetically modified bacteria for the hydrolytic fermentation are under development, in order to overcome all the issues related to the high cost of the production processes.

Finally, in this work it was reported that wood waste can be used not only as a source for the production of third-generation biopolymers, but it can also be employed as a reinforcement for bio-based matrices, thus obtaining biocomposites with improved mechanical performances, as well as enhanced antibacterial, gas barrier and migration properties. Therefore, following both a descriptive and an analytical methodology, the main properties of bio-based polymers and biocomposites were discussed in this review and a comparison of thermal and mechanical properties of polymer matrices and wood biocomposites was reported.

## Figures and Tables

**Table 1:** A brief definition and classification of bio-based polymers.

Types	Chemical Group	Example	References
Biomass-based polymers	Polysaccharides Proteins and lipids	Carbohydrates (wheat, potatoes, maize); Products made from cellulosic and ligno-cellulosic materials (wood, straws, etc.); Pectins (chitosan/chitin, gums, etc.); Casein, whey, collagen/gelatin from animals; Plants which are a type of living thing (zein, soya, gluten).	[16,17,18]
Polymers derived via microbial fermentation	Polyhydroxyalkanoates (PHA)	Poly(hydroxybutyrate) (PHB) Poly(hydroxybutyrate-cohydroxyvalerate) (PHBV)	[19,20,21]
Agro-resource monomers are used to chemically manufacture polymers	Poly(hydroxyacid)	Poly(lactic acid) (PLA) Polyglycolic acid (PGA)	[22,23,24]

**Table 2:** Definition of compostability and biodegradability of some bioplastics in different environments.

Bioplastic	Biodegradability					Ref.
	Environment	Condition	Biodegradability (%)	Biodegradability/ Degradability Method	Testing Period (Day)	
Starch-based	Compost (starch, thermoplastic)	58 °C	73.1	CO_2_ produced	56	[11,27,28,29]
	Soil (wheat, starch-derived plastic)	20 °C, 60% RH	14.2	CO_2_ produced	110	
	Marine (neat starch)	26 °C	100	Weight loss	50	
Cellulose-based	Compost (cellulose acetate)	53 °C	100	CO_2_ produced	18	[11,27,28]
	Soil (bacterial and vegetable cellulose)	25 °C	100	Weight loss	180	
	Simulated marine environment (neat cellulose)	Room temperature	75	Oxygen consumed	150	
PLA	Compost	58 °C, 60% RH	60–70	CO_2_ produced	30	[11,12,27,30]
	Soil	10–25 °C	0	CO_2_ produced	120	
	Simulated marine environment	25 °C	3–4	CO_2_ produced	180	
PHB	Compost	55 °C, 70% RH	80	CO_2_ produced	28	[11,27,28]
	Soil	20 °C, 60% RH	48.5	CO_2_ produced	280	
	Simulated marine environment	25 °C	38–45	CO_2_ produced	180	

**Table 3:** Young’s modulus (MPa) in relation to the added plasticizer and the initial polymer structure.

	Young’s Modulus	Amorphous Plasticized PLA (MPa)	Semicrystalline Plasticized PLA (MPa)
Plasticizer			
PEG 400		581	962
Cardanol		691	1156
Epoxidized cardanol		353	961

**Table 4:** Some features of the main types of bio-based wood composites.

Bio-Based Polymers	Density (g/cm^3^)	Tensile Strength (MPa)	Young’s Modulus (GPa)	Elongation at Break (%)	Tg (°C)	Tm (°C)	Ref.
Wood–starch	1.29–1.41	14–36	0.7–4.8	1.1–2.9	−33–−26	83.1–130.3	[161,169,176,177,180,181,182,183,184]
Wood–PLA	1.26–1.41	30–71	1.2–8.9	1.0–3.1	52.0–60.8	143.4–169.0	[12,154,161,162,163,181,185,186,187,188,189]
Wood–PHA	1.23	13–65	0.4–6.1	0.5–7	−2.2–−6.0	56.8–158.7	[161,170,172,190,191]

**Table 5:** Main properties and applications of some bio-based polymers and biocomposites.

Bio-based Polymers and Biocomposites	Properties	Applications	Source	Ref.
Starch-based	Low toxicity, biocompatibility and equivalent mechanical and degrading qualities.	Packaging applications, wound-healing materials, drug delivery system, agricultural foils, textiles, automobiles and transportation, construction and building materials, etc.	Plants	[215,261,265,266,267]
Cellulose-based	Microbial characteristics, exceptionally crystalline, chemically and thermally stable.	Packaging applications, edible films and coatings, hydrogels for personal care products, medical device, biosensors, drug delivery system, electronic and energy devices, cosmetics, textiles and nonwovens etc.	Plants	[45,250,256,259]
Lignin-based	Biodegradability, hydrophilicity, low-cost, nontoxicity, thermal and mechanical stability	Food packaging, applications in biocide systems, adhesive resins and foams, filling materials, construction and building materials, biomedical applications	Plants	[154,205,227,231,268]
PLA-based	Higher mechanical strength, degradation in nature either through reduction or by M, excellent barrier and permeability properties.	Packaging applications, 3D printing, biomedical applications, scaffolds and medical implants, textiles and nonwoven fabrics, agricultural applications, etc.	Fermentation/conventional chemistry followed by polymerization	[12,226,238,264,269]
PHA-based	Biocompatible, biodegradable, considerable elastomeric with excellent elongation at break.	Food packaging and coatings, scaffolds and medical implants, textile industry, etc.	Bacterial fermentation	[205,207,270,271]

## Data Availability

The data presented in this study are available on request from the corresponding author.
